# Experimental study of an asymmetric valveless pump to elucidate insights into strategies for pediatric extravascular flow augmentation

**DOI:** 10.1038/s41598-022-26524-0

**Published:** 2022-12-22

**Authors:** J. Anatol, M. García-Díaz, C. Barrios-Collado, J. A. Moneo-Fernández, M. Horvath, T. Parra, F. Castro-Ruiz, E. T. Roche, J. Sierra-Pallares

**Affiliations:** 1 Departamento de Ingeniería Energética y Fluidomecánica and ITAP, Universidad de Valladolid, Paseo del Cauce 59, 47011 Valladolid, Spain; 2 Institute for Medical Engineering and Science, Massachusetts Institute of Technology, Cambridge, MA 02139 USA; 3 Department of Mechanical Engineering, Massachusetts Institute of Technology, Cambridge, MA 02139 USA

**Keywords:** Biomedical engineering, Mechanical engineering

## Abstract

Asymmetric pumping is a sub-category of valveless pumping in which a flexible tube is rhythmically compressed in the transverse symmetry plane. Due to the resulting asymmetry between the suction and discharge pipes, a net pumping head is achieved. Asymmetric pumping is regarded as one of the main mechanisms responsible for the Liebau effect in addition to impedance pumping. However, there remains a paucity of research surrounding the governing parameters of asymmetric pumping. Here, we conducted an experimental study of the performance of an asymmetric pump, with an aim to assess its potential for extravascular flow augmentation. A custom flexible latex tube and experimental platform were developed for this purpose. We tested various tube thicknesses and pinching frequencies. Our results demonstrate that the performance is within the range of physiological requirements for pediatric circulatory devices (~ 1 L/min and < 30 mmHg). We conclude that due to the absence of reverse flow and its mechanical simplicity, pure asymmetric pumping is promising for selected cardiovascular applications with less complexity than other valveless techniques.

## Introduction

The category of valveless pumping encompasses phenomena which can generate a controlled, unidirectional flow without valves. One of the most widely studied valveless pumping mechanisms is the Liebau effect^1^. A Liebau pump is a small device consisting of a straight elastic tube with two differentiated segments: a wider and more distensible segment, and a narrow, stiff segment. Upon cyclical compression of the wider segment, a net flow is achieved towards the narrower segment. The Liebau effect can be explained as the superposition of two different pumping mechanisms: impedance pumping and asymmetric pumping^2^.

Impedance pumping occurs in a circuit where a compliant tube is connected to rigid pipes creating a sharp difference in impedance. Due to this, the pressure waves are strongly reflected in both compliant tube ends. Additionally, the pincher is not placed at the compliant tube symmetry plane. As a result of the pinching, mechanical energy is added to the fluid, mainly in form of pressure. These pressure waves travel from the pinching region towards both ends of the compliant tube, where they are reflected. As the pincher is not equidistant from both ends, the reflected pressure waves do not cancel each other, generating a pressure field that creates the pumping effect^3^.

**Figure 1:**
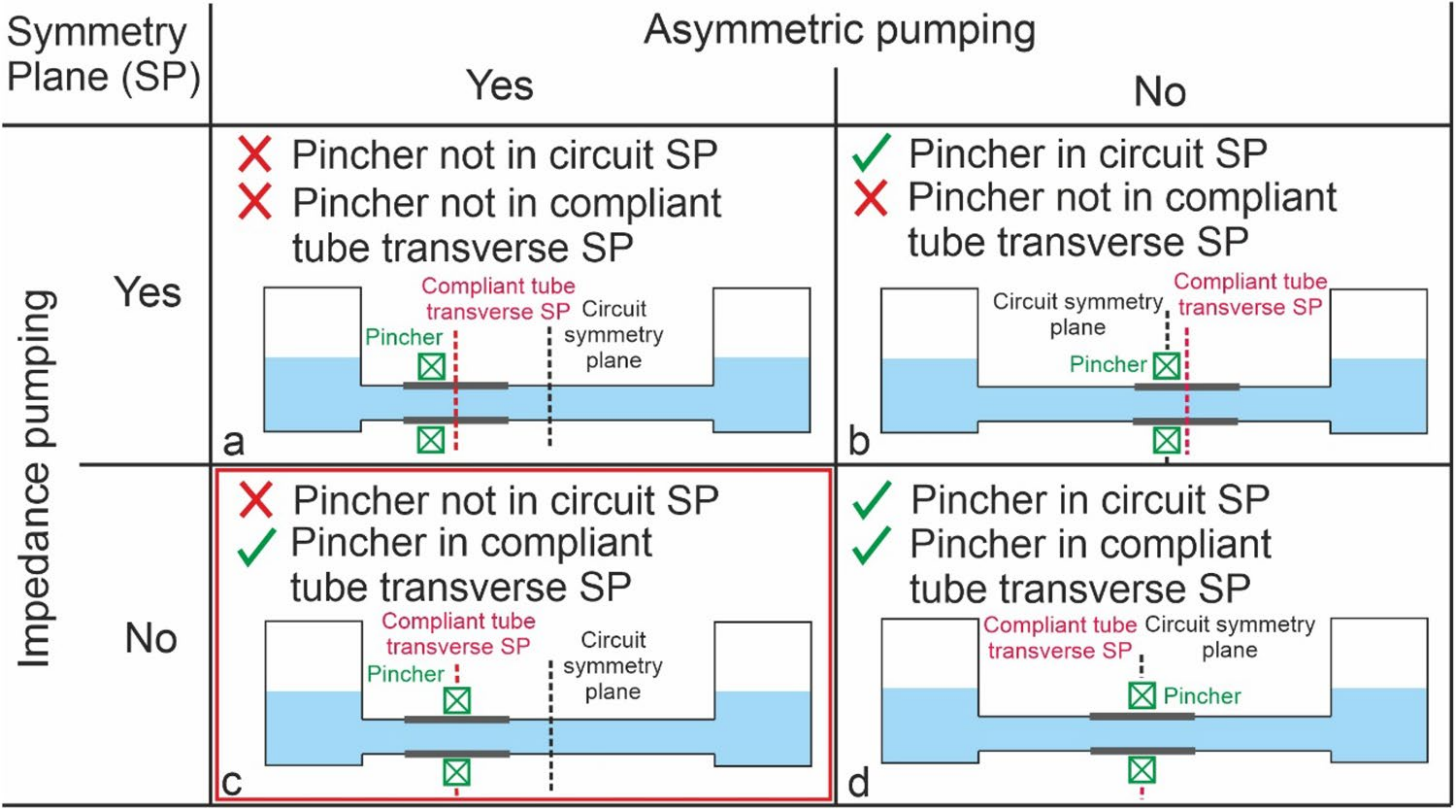
Impedance and asymmetric pumping illustrating asymmetry through length of compliant tubing. (**a**) Mixed (Liebau effect), (**b**) impedance, (**c**) asymmetric and (**d**) no pumping systems. SP = symmetry plane.

Conversely, in asymmetric pumping the actuator is located at the compliant tube symmetry plane and thus reflected pressure waves cancel each other. The main feature of asymmetric pumping is that an asymmetry in the hydraulic resistance of the rigid pipes is necessary to achieve pumping. In order to illustrate this classification, Fig. 1 considers length as the only asymmetric parameter, and includes an example that would not achieve pumping (Fig. 1d). More broadly, asymmetry could be achieved by varying the diameter, material or design of the compliant tube. In all cases, the pumping effect is generated by pulse pressure waves travelling through the system. Asymmetry results in a net axial pressure gradient^4^.

Since its discovery in the mid-twentieth century, numerous studies have described the mechanism of the Liebau pump—a mechanically simple device with a dynamically complex mechanism. The practical applications of the Liebau pump are diverse, from cardiovascular assistance to electronics. To parse the fundamental principles governing this phenomenon, Hickerson et al. ^5^ experimentally demonstrated the sensitivity of Liebau pumping to various parameters including changes in pincher position, size and compression frequency, transmural pressure, systemic resistance and materials in a closed loop. Hickerson and Gharib^6^ continued these experiments to show the wave mechanics required for the build-up of pressure and net flow. Ultrasound measurements of the transient and resonant properties were used to relate bulk responses to pump mechanics. Wen et al.^7^ carried out an experimental study of a Liebau pump for the thermal management of high performance electronic systems, showing its feasibility for electronic cooling. From an analytical and computational modelling perspective, several studies have examined the phenomenon^8–11^, often using one-dimensional approaches to solve momentum and mass balances, and comparing experimental and analytical results. Notably, Avrahami and Gahrib^4^, conducted a complete fluid-structure interaction simulation of a Liebau pump, which comprehensively explained the physical phenomena underlying wave pumping. From the application perspective, Pahlevan and Gharib^2^ performed an in vitro investigation of a potential wave pumping effect in the human aorta, showing the difference in asymmetric and impedance pumping, and concluding that wave propagation and reflection can result in a pumping mechanism in a compliant aorta. Recently, Davtyan and Sarvazyan^12^ confirmed the physiological feasibility of Liebau-based pumping in an experimental set-up using anatomically-sized vessels, demonstrating pumping performance comparable to that of similarly sized peristaltic pumps.

Although several studies deal with the Liebau pump and its potential applications, there is less research elucidating the main mechanisms of pumping and subsequent application to cardiovascular assistance. In the present study we address this gap with a customized modular experimental setup, in which the Liebau effect phenomena can be studied, enabling a highly controllable experimental environment. It includes dimensions, flowrates and materials that operate within the range of parameters likely to be encountered in cardiovascular pathophysiology. Additionally, this paper shows the performance of a completely new pincher, based on the mechanical concept of a diaphragm shutter, to enable a much more uniform pinching that allows a better theoretical performance than flat type pinchers^4^.

This paper is organized as follows; first, we explain our material and methods, including the manufacturing and validation of our custom flexible latex tubes and the asymmetric pumping test bench. Second, we present the results of pumping performance as a function of latex tube compliance, head versus flowrate curves and the effect of compression frequency on the pincher.

## Methods

To conduct this experimental study, we built two experimental setups; the first to characterize custom-manufactured latex tubes, and the second hydraulic circuit to determine the performance of the asymmetric pump. We established procedures to ensure the repeatability and accuracy of the tests.

### Manufacturing the custom latex tube

**Figure 2:**
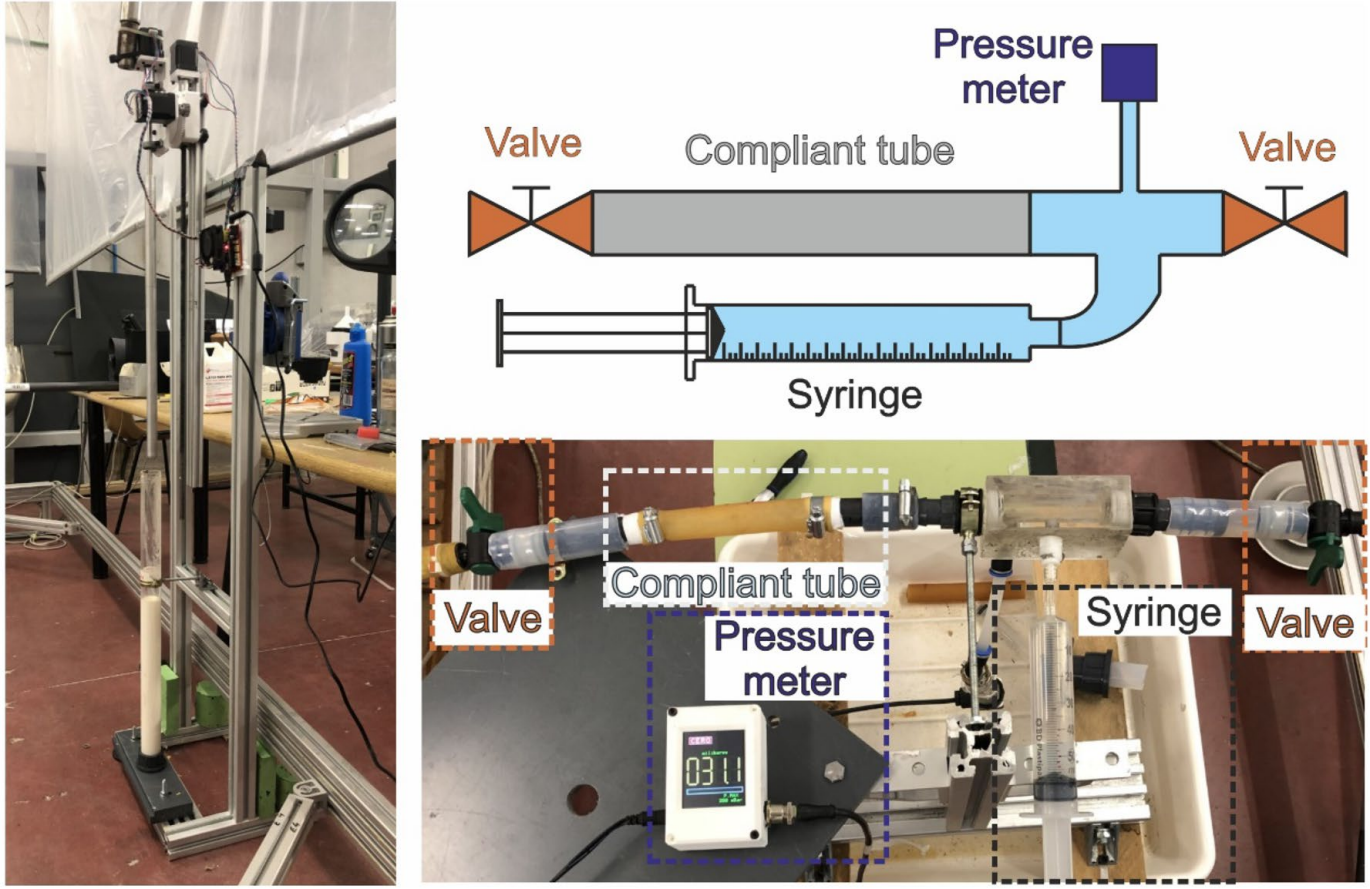
Latex tube manufacturing station (left) and compliance measurement setup (right).

Our latex tubes were custom-manufactured in our facility, Fig. 2. A rotating aluminum bar (3 rpm, 40 cm long, 2 cm in diameter) was vertically submerged inside an ammonia-based liquid latex (Feroca, Spain) in a glass container. Next, it was extracted to cure the adhered latex layer. This process was repeated several times to achieve the desired wall thickness (ranging from 0.3 mm to 1.05 mm), taking a few hours to complete. The process was completely automated for reproducibility. The setup is controlled by an Arduino Nano, which is programmed with a custom program for each thickness of the latex tube.

The result of the process was a 40 cm long, cylindrical, latex casing around an aluminium mandrel, which was carefully removed once the latex was dry. After the ends were removed, the tube was cut into three identical latex tubes, each 12 cm long. The compliance, C, of the latex tubes was measured experimentally. Compliance is defined as per Eq. (1)^13^,

1 $$C = \frac{{\partial {\text{V }}/\partial {\text{P }}}}{{{\text{Vo}}}}$$

where V, P and V_0_ are, respectively, volume, pressure and tube volume at rest conditions (V_0_ = 31.4 mL). Compliance was measured by injecting known volumes of tap water through a syringe inside a latex tube closed at both ends and measuring the transmural pressures; with a pressure meter (JUMO dTRANS p30, Germany), as shown in Fig. 2. Injected volumes and read-out pressures measured up to 20 mL and 250 mbar, respectively.

### Experimental pure asymmetric pumping test setup

**Figure 3:**
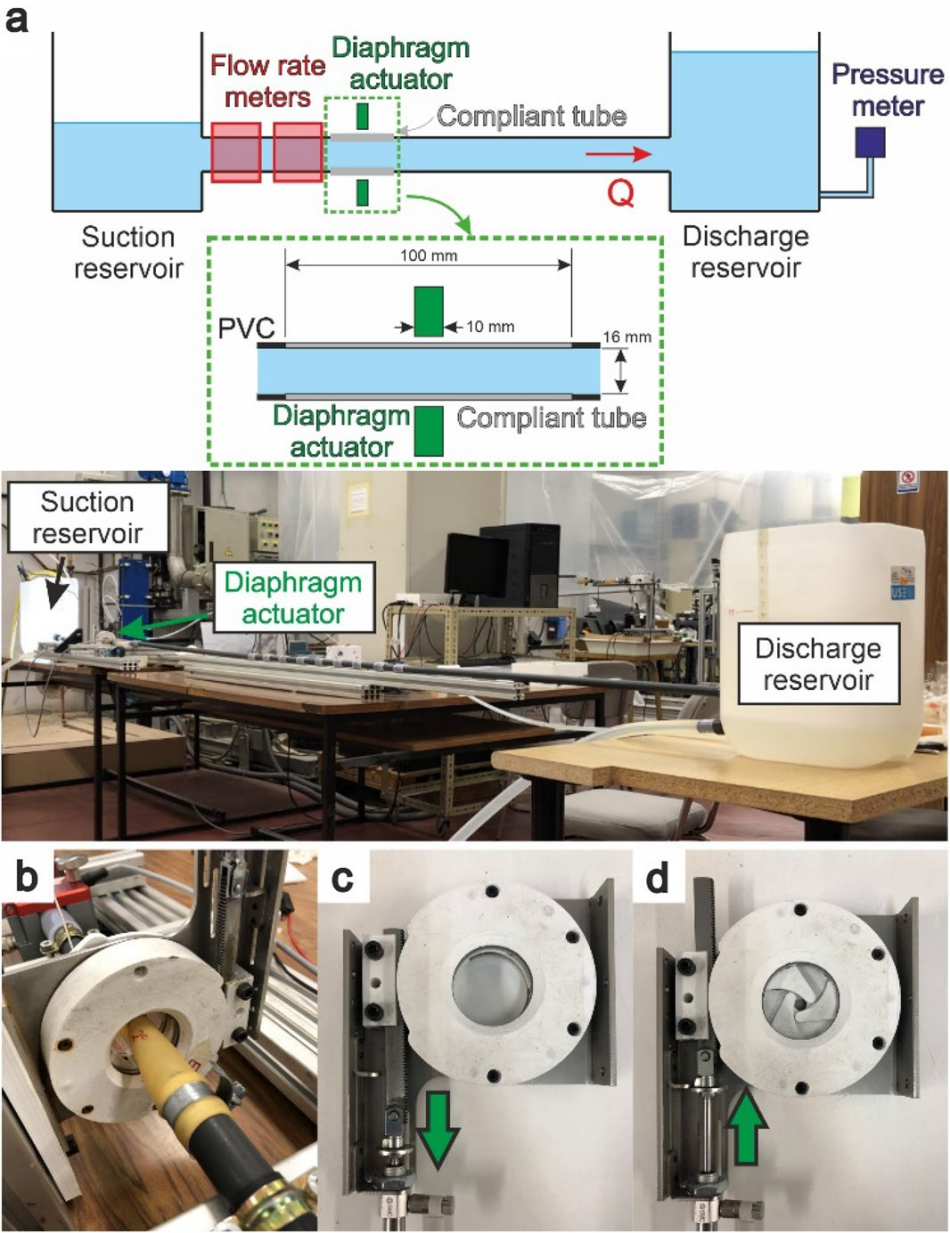
(**a**) Pure asymmetric test rig with λ = 4.33 (**b**) diaphragm shutter actuator mounted in the test rig, (**c**) diaphragm shutter open, (**d**) diaphragm shutter closed.

Our experimental asymmetric pumping test circuit (Fig. 3a) consisted of two large reservoirs (750 cm^2^ cross-sectional area) 4 m apart, connected via a straight, horizontal, 20 mm outer diameter, 16 mm inner diameter tube. The tube was made of non-compliant pipe, except for a 10 cm long latex portion and two small silicone tubes, one on each side of the latex tube, enabling flow measurement with two unidirectional ultrasound flowmeters (Sonotec Sonoflow CO.55/230H V2.0, Germany). The test circuit was equipped with a pressure meter (Keller PD-23, Switzerland) in the discharge reservoir, to enable measurement of the height of the water in the reservoir. Uncertainties regarding the flowmeters, the pressure meter and compliance were approximately 2%, 4% and 0.5%, respectively. Uncertainty for period/frequency was up to 0.025%. The discharge-to-suction pipe length ratio λ = 4.33. The system is conceived as a platform to focus on the study of asymmetric pumping, able to study very different types of assistance.

We used a 3D printed, pneumatically driven, six-blade diaphragm shutter as a pincher, placed concentrically around the latex tube at the compliant tube transverse symmetry plane (Fig. 3b–d). The closure design was an improvement on the traditional method of flat plate compression in experimental studies^5^. The radial closure of two planes with three blades each resulted in a 10 mm wide compression region, Fig. 3d. The ratio A_b_^9^ is defined in Eq. (2)

2 $$A_{b} = \frac{{A_{0} - A_{min} }}{{A_{0} }}$$

where A_0_ is the undeformed tube cross-sectional area and A_min_ is the minimum shutter cross-sectional area. A_b_ was set to 65% to prevent the complete occlusion of the latex tube, Fig. 3d.

The compression duty cycle^2^ was fixed at 33%. Frequency was either manually controlled or automatically adjusted. In manual mode, frequency was set at a fixed value for the entire test. In automatic mode, the system itself updates pinching frequency to an optimized value referred to as “resonant frequency” as performance peaks in such conditions and falls abruptly outside it^14^. In order to adjust this value, the compression was triggered at the time when the flowrate wave arrives at the pincher, after being reflected at the suction reservoir. Thus, the control system can self-update the working frequency during the tests to achieve optimal performance. Tested frequencies were in the range of < 5 Hz, with a time resolution of one millisecond. The Womersley number (Wo) was approximately 13, based on Eq. (3)

3 $$Wo = R \sqrt {\frac{\rho f}{\mu }}$$

where R, ρ, f and µ are vessel radius, density, frequency and viscosity, respectively.

Assuming a representative flowrate of 1 L/min in the setup, we calculate a Reynolds number (Re) of approximately 1000, which corresponds to the laminar flow regime. We therefore estimated a maximum head loss^15^ of less than 2 mm. Since the height difference observed between the free surfaces of the reservoirs was in the centimeter range, we used this as pump head and neglected losses. Initially (when the system is at rest and the pressure head is equal to zero), both reservoirs were at a height of 15 cm above the tube circuit. Tap water was used as the working fluid, and special care was taken to purge air from the test rig.

The homebuilt data acquisition system was based on an Arduino Due system and sampled data from both the pressure meters and the flowmeters every 1 ms.

**Figure 4:**
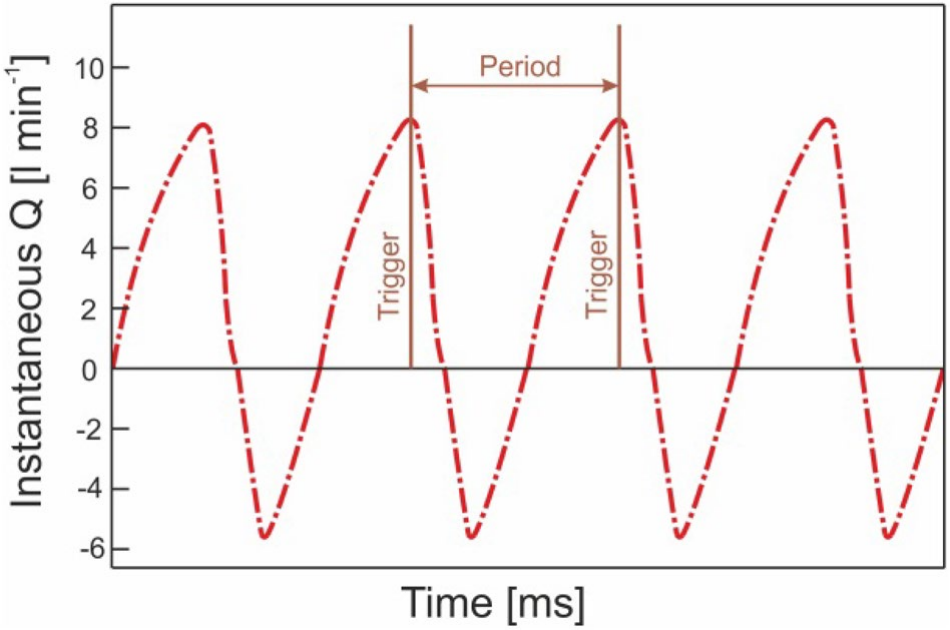
Software trigger for triggering closure of the pincher.

For the control system, an Arduino Nano controlled the diaphragm shutter closure; this can be set in two different modes. In manual mode, a constant work frequency is fixed for the entire test. In automatic mode, the control system determines the resonant frequency from the signal of one of the flowmeters and as such synchronises diaphragm shutter closure with the refilling of the latex tube. In all tests, unless otherwise stated, we used the automatic mode. Figure 4 shows the flowrate time course that the control system employs to set the closure trigger from the peak of the flowrate curve. In this case, positive flowrate is from the suction to the discharge reservoirs. The period is calculated based on the time between consecutive peaks.

### Data analysis

Data was processed and analyzed with MATLAB. The recorded time series data of instantaneous head and flowrate were filtered with a moving average filter in order to reduce noise. To extract head vs flowrate curves, values were averaged for each compression cycle. For characterization of performance, both head and flowrate were considered—head was time-asymptotic in terms of its maximum value^16,17^ while the maximum flowrate occurred at the time of the first compression cycle, making it difficult to capture with ultrasound flowmeters. As head is approximately linear with time during the first part of experiment (approximately 60 cycles), the maximum flowrate was calculated as the pumped volume over time for the first 5 cycles^16^.

## Results

### Compliance tests

**Figure 5:**
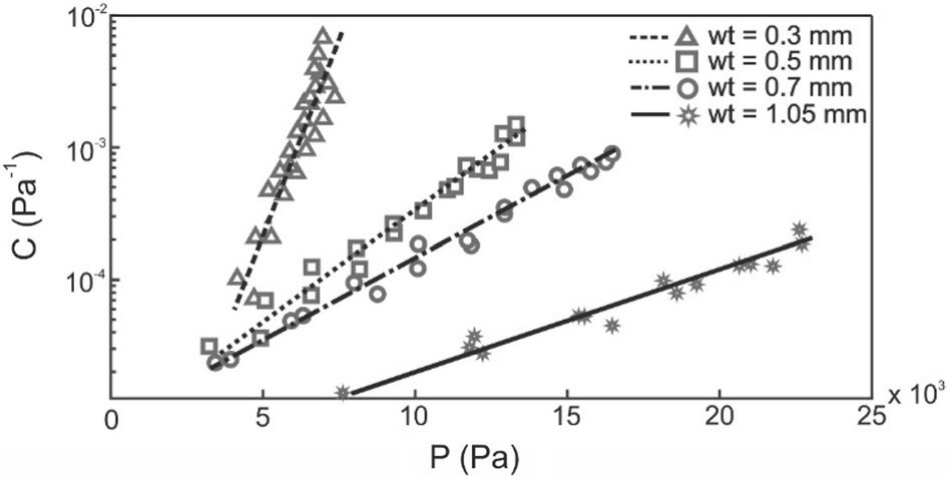
Compliance test of several sets of tubes of different wall thicknesses: 0.3, 0.5, 0.7, 1.05 mm.

Figure 5 shows the measured tube compliance for several sets of latex tubes with varying wall thickness (w_t_). Compliance is plotted on a logarithmic y-axis. Our custom latex tubes show good repeatability for each wall thickness, suggesting that the properties along the length of the tube are homogeneous and that our production method is consistent. Compliance follows an exponential relationship with pressure. We hypothesize that such a clear rising tendency is due to the fact that, as volume increases, wall thickness becomes thinner and thus the latex tube behaves in a less rigid manner. In our subsequent experiments, transmural pressure ranged from 500 to 3000 Pa, in this pressure range compliance is similar for all wall thicknesses (Fig. 5).

### Pump performance at resonant frequency

**Figure 6:**
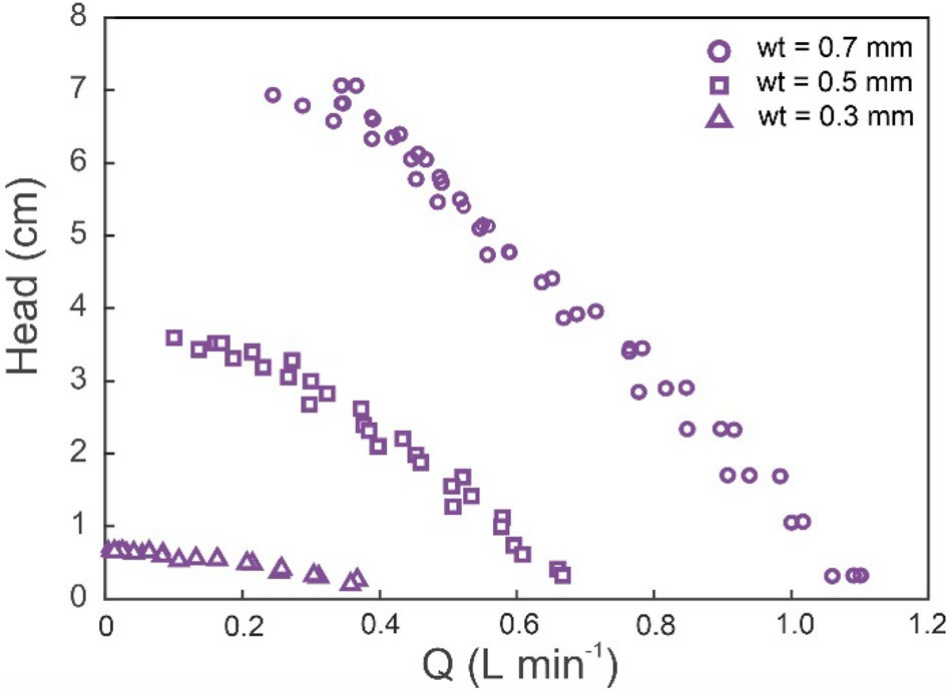
Head versus flowrate curves for several latex tube wall thicknesses (wt).

Immediately after their compliance was measured, the latex tubes were installed on the test rig to ascertain their characteristic pump curves. Figure 6 shows head versus flowrate curves for several latex tubes of different wall thicknesses, with compression at the resonant frequency (roughly 1.72 Hz, or 103 BPM). These curves show good repeatability. It can be appreciated that greater wall thickness improves pumping performance. All latex tubes were used immediately following compliance testing, and their durability was limited to ~ 5,000 compression cycles. In this period, no significant change in pumping performance was observed, except at the time of failure. Since, for these tests, the control system calculated the resonant frequency every compression cycle, this changed during the experiment. In the case of the 0.7 mm thick latex tube, a progressive frequency increase of approximately 5% was observed as the head rose. Nevertheless, changes in resonant frequency were negligible for latex tubes with a thickness of 0.3 and 0.5 mm.

**Figure 7:**
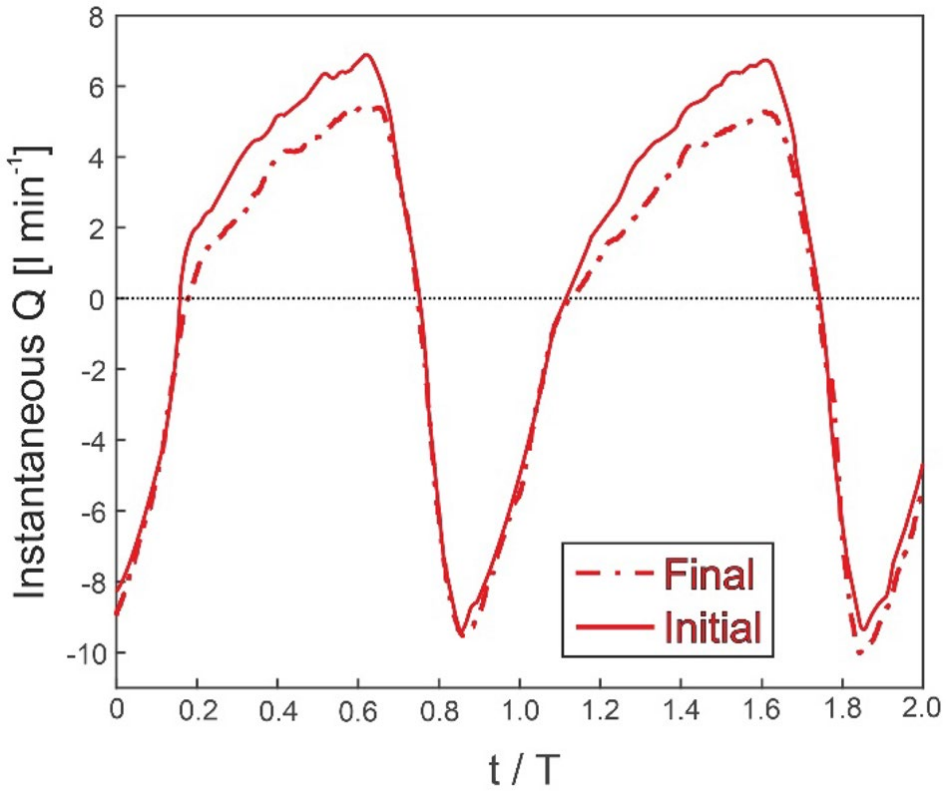
Instantaneous flowrate for the initial and final parts of the tests.

Instantaneous flowrate was studied in order to aid with understanding the phenomenon of asymmetric pumping. Figure 7 shows the instantaneous flowrate at the suction tube for both the initial and final experiment (0.7 mm thickness, resonant frequency). Initially, the head was zero (both reservoirs had the same volume of liquid) and the pump delivered its maximum flowrate. The instantaneous flowrate displayed a waveform influenced by the compression pattern. Negative flowrates correspond to fluid displaced from the pincher to the suction reservoir. They occur when the diaphragm shutter closes and displaces the water inside the latex tube to both ends. Positive flowrates correspond to the incoming flowrate wave which refills the latex tube. The net (or cycle-averaged) flowrate is the difference between the positive and negative parts of the instantaneous flowrate wave, Fig. 7. Both positive and negative flows peak around one order of magnitude higher than the maximum net flowrate. The pattern of the instantaneous backflow seems to remain constant; however, there is a difference between the initial and the final positive parts of instantaneous flowrate waves. Accordingly, the positive pumped volume decreases as head increases during the experiment. Positive and negative volumes are calculated as the area inside the positive and negative flowrate curve and the x-axis, respectively. When the maximum head is reached, positive and negative volumes are equal. Consequently, net flowrate is zero.

### Frequency spectrum

**Figure 8:**
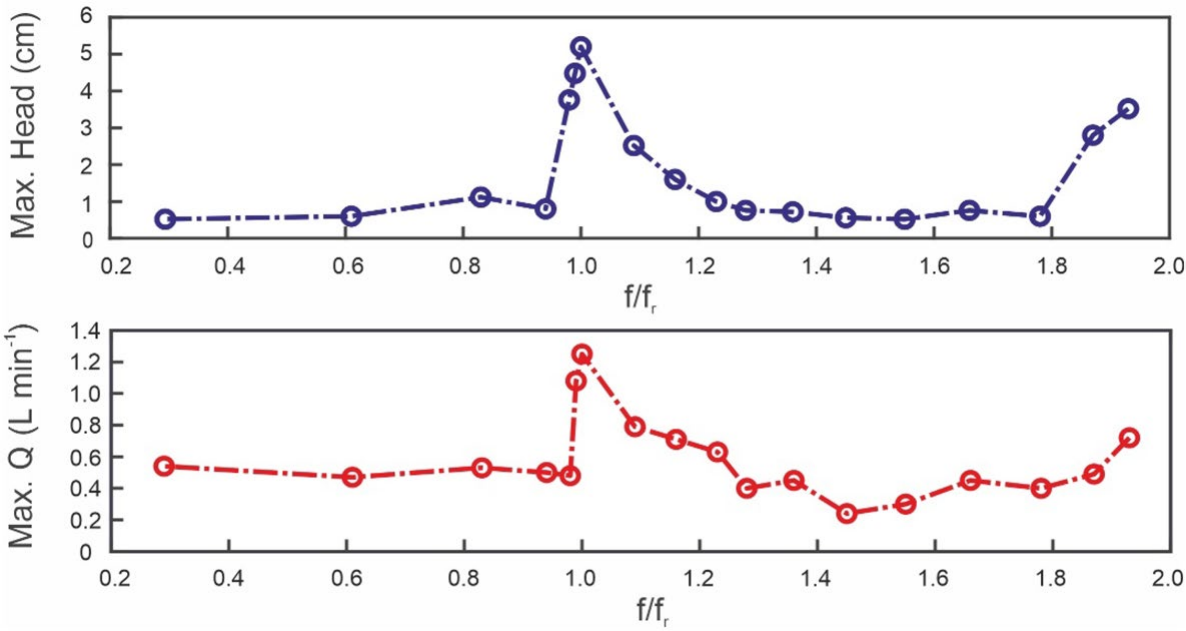
Maximum head and maximum net flowrate frequency spectra.

An experimental series was run at fixed compression frequency ($f$), ranging from frequencies low enough for no significant performance to be observed to approximately twice the resonant frequency (${f}_{r}$), with 0.7-mm-thick latex tubes. An upper frequency limit was imposed by mechanical limitations of the test rig, mainly the actuator and the durability of the latex tubes. Figure 8 shows the maximum head and maximum net flowrate frequency spectra. Clearly, there is a nonlinear relation between the net flow rate and frequency. Good performance at the resonant frequency can be observed, although there is an abrupt drop with small variations in frequency. The trend reveals that there is another significant performance peak at the second harmonic, similar to other frequency-dependent phenomena. Despite poor performance outside the resonant peak performance, we did not observe net reverse flow, i.e., no negative heads or flowrates.

## Discussion

Pure asymmetric pumping has already been assessed in the literature, by means of a piston pump^8,18^, rather than a combination of a flexible tube with an external pincher. The configuration of a piston pump is unsuitable for extravascular applications, as it requires blood contact with the actuator. Most research regarding valveless pumps based on compressing flexible tubes focuses on the Liebau effect, which requires pincher asymmetry. Moreover, a large number of studies mix both the effect of asymmetric compression and asymmetric suction and discharge geometries^17^, which can add complexity.

In this study, we provide insights into an asymmetric valveless pumping effect, where, although the flexible element is symmetrically compressed, it is positioned non-symmetrically in the circuit system (Fig. 1c). We focus on overall experimental performance (i.e., period-averaged head and flowrate). According to this configuration, and based on the existing literature on the subject, we compared our results with the the period—averaged mechanical energy equation described by Propst^11^:

4 $$\overline{h}_{D} - \overline{h}_{S} = \frac{1}{2g}\left( {\zeta_{D} \overline{w}_{D}^{2} - \zeta_{S} \overline{w}_{S}^{2} } \right)$$

where ${\overline{h} }_{D}$ represents the period averaged level of the discharge reservoir, ${\overline{h} }_{S}$ the same magnitude for the suction reservoir, ${\zeta }_{D}$ and ${\zeta }_{S}$ are the head loss coefficients for the discharge and suction parts of the circuit and ${\overline{w} }_{D}$ and ${\overline{w} }_{S}$ are the mean period averaged velocities in each part of the circuit. In our experiments, this difference is always positive in agreement with the period averaged energy balance. Water flows from suction to discharge reservoir due to the difference in kinetic energy of both branches, as pointed out elsewhere^4,11^. Additionally, working at the resonant frequency as outlined above, agrees with the theoretical work of Jun and Kim^19^. The phase synchronization in time between the fluid pressure difference and the external pinching force is obtained when working at the resonant frequency, thus enabling the energy storage in the discharge reservoir.

Transmural pressures under working conditions (between 500 and 3000 Pa) were far lower than those measured by our compliance test bench. Therefore, according to Fig. 5, tube compliance under working conditions for the three thicknesses is represented by similarly low values. Consequently, it is hypothesized that under such conditions, differences in tube compliance play a negligible role in terms of resonant frequency. Since everything else remains constant, resonant frequency should not change. We maintain that the slight drift observed on the resonant frequency towards higher values as the head increases is related to the superior performance of the 0.7 mm thickness tube. Moreover, latex tube thickness seems to play an important role in performance, as thicker tubes are associated with higher heads and flowrates. Our hypothesis is that stiffer tubes displace larger volumes, whereas more flexible tubes may dilate outside the pinching region, damping the compression effect and thus displacing smaller volumes.

Maximum head and flowrate performance were observed at a particular frequency, referred to as resonant frequency ${f}_{r}$ (or multiples of this frequency). This observation is also found in the literature^5,6^ for impedance pumping, where it is related to the pressure wave speed (c) and the length of the flexible tube suction side (L), as in Eq. (5).

5 $$f_{r} = \frac{c}{2 L}$$

However, the wave speed cannot be calculated by means of the well-known Moens-Korteweg equation, which overestimates it by an order of magnitude^12^. Although some authors^9,13^ propose other improved analytical approaches or variations of the Moens-Korteweg equation, approximation errors are still far from acceptable given the narrow resonance peaks.

Flow direction in our open-loop, atmospheric test rig configuration seemed not to depend on frequency, as no reverse flow was observed in the frequency sweep conducted. Although frequency-dependent flow direction in asymmetric valveless pumping has been reported^2,8,18^, this may be the result of significant differences in geometry and boundary conditions such as closed loops or piston actuators. Determining under which boundary conditions unidirectional flow is achieved would be of vital importance for extravascular assistance applications.

Future work will address the effect of varying the overall tube length, the discharge-to-suction length ratios, and the suction head on the resonant frequency and performance. For extravascular pumping assistance, the valveless pump would be coupled to a larger system working under variable conditions^14^. The narrow resonant peak bandwidth highlights the critical importance of compressing at the resonant frequency. We achieved an online frequency setting by means of an ultrasound flowmeter. However, other less invasive sensing techniques could be developed for clinical applications. Optical sensors (such as wrist-based heartrate monitors) or embedded pressure gauges seem may be promising techniques. The asymmetric pump displayed successful pumping by increasing head pressure and flowrate. The current embodiment is able to pump in a range suitable for pediatric circulatory support with pressures of < 30 mmHg (around 41 cm H_2_O) and flowrates of around 1 L/min which correspond to physiological conditions^20^. The operational parameters and embodiment of the pump will be further refined and optimized to address different hemodynamic conditions in the future. The resonant frequency is physiologically compatible (0.5–2.5 Hz)^12^.

In conclusion, the study suggests that pure asymmetric pumping is a promising technology for use in extravascular flow augmentation devices. Although refinement is required before clinical translation, the preliminary results are encouraging and the technique has considerable advantages over alternative valveless pumping techniques including peristaltic and impedance pumping. Asymmetric pumping is mechanically simpler than the other options and does not induce reverse flow at physiological compression frequencies, an important consideration for use in extravascular blood flow augmentation.

## Data Availability

Experimental data will be available on request, please contact the corresponding author at jsierra@eii.uva.es.
